# Behavioral effects of adult male mice induced by low-level acetamiprid, imidacloprid, and nicotine exposure in early-life

**DOI:** 10.3389/fnins.2023.1239808

**Published:** 2023-08-16

**Authors:** Hirokatsu Saito, Yusuke Furukawa, Takahiro Sasaki, Satoshi Kitajima, Jun Kanno, Kentaro Tanemura

**Affiliations:** ^1^Division of Cellular and Molecular Toxicology, Center for Biological Safety and Research, National Institute of Health Sciences, Kawasaki, Japan; ^2^Laboratory of Animal Reproduction and Development, Graduate School of Agricultural Science, Tohoku University, Sendai, Japan

**Keywords:** neonicotinoid, central nervous system, neurobehavioral effect, low-level exposure, developmental neurotoxicity

## Abstract

**Introduction:**

Acetamiprid (ACE) and imidacloprid (IMI), the neonicotinoid chemicals, are widely used as pesticides because of their rapid insecticidal activity. Although these neonicotinoids exert very low toxicity in mammals, the effects of early, low-level, chronic exposure on the adult central nervous system are largely unclear. This study investigated the effects of low-level, chronic neonicotinoids exposure in early life on the brain functions of adult mice, using environmentally relevant concentrations.

**Methods:**

We exposed mice to an acceptable daily intake level of neonicotinoids in drinking water during the prenatal and postnatal periods. Additionally, we also exposed mice to nicotine (NIC) as a positive control. We then examined the effects on the central nervous system in adult male offspring.

**Results:**

In the IMI and NIC exposure groups, we detected behavior that displayed impairment in learning and memory. Furthermore, immunohistochemical analysis revealed a decrease in SOX2 (as a neural stem cell marker) and GFAP (as an astrocyte marker) positive cells of the hippocampal dentate gyrus in the IMI and NIC exposure groups compared to the control group.

**Discussion:**

These results suggest that exposure to neonicotinoids at low levels in early life affects neural circuit base formation and post-maturation behavior. Therefore, in the central nervous system of male mice, the effects of low-level, chronic neonicotinoids exposure during the perinatal period were different from the expected effects of neonicotinoids exposure in mature animals.

## 1. Introduction

Since various chemical substances exist in the environment, organisms are always exposed to some kind of chemical substance. Therefore, chemical substances are considered one of the typical environmental stresses. Although the risk assessments are necessary to prevent the health hazard induced by chemical exposure and are updated, several severe harmful effects on brain functions are yet to be identified. Recently, the chemical exposure of mammals in the prenatal, early postnatal, or juvenile periods has been recognized as a potential inducer of multiple effects on the nervous, immune, and reproductive systems in adulthood, even when exposed at low levels (United Nations Environment Programme and World Health Organization, 2013). In particular, exposure to pesticides has raised concerns about their effects on children’s brains, even low-level (Roberts et al., 2019). In the previous study, we demonstrated the possibility that exposure to low-dose chemicals in early life affects the central nervous and reproductive systems after maturity (Saito et al., 2017, 2019). In the results of this study, we indicated the importance of carefully considering regulatory values for prenatal, early postnatal, and juvenile individuals for chemical substances that are suspected to affect mammals.

Neonicotinoids are a new class of insecticides chemically similar to nicotine (NIC). These are modeled after the chemical structure of NIC and have been used as pesticides in recent years. They have been considered to have low toxicity to mammals because they have a much higher affinity for the nicotinic acetylcholine receptors (nAChRs) of insects than mammals (Tomizawa and Casida, 2005). However, neonicotinoids have been reported to have nicotine-like excitatory effects on nAChR of mammals (Kimura-Kuroda et al., 2012), and there is concern that exposure to neonicotinoid pesticides may have adverse effects on the central nervous system of mammals as behavioral abnormalities (Abreu-Villaca and Levin, 2017). NIC and nAChRs are closely associated with cognitive behavior like learning and memory (Herman and Sofuoglu, 2010). Several studies indicate that prenatal nicotine exposure has adverse effects on neurodevelopment and behavior, even at doses that do not affect survival or apparent growth (Navarro et al., 1989; Eppolito and Smith, 2006). Therefore, neonicotinoids can also affect mammalian brain functions such as cognitive behavior. In the developing brain, various neuronal signals must be activated appropriately to construct the neural network, and exposure to chemical substances in this period can induce developmental neurotoxicity (Rice and Barone, 2000). Therefore, exposure to neonicotinoids in early life disrupts neural signals, irreversibly affects the developing brain and leads to behavioral effects after maturation. Exposure to neonicotinoid pesticides during the prenatal and early postnatal periods causes neurobehavioral impairments, including anxiety behavior, impairment of learning and memory, and deviation of sociality (Ozdemir et al., 2014; Kara et al., 2015; Sano et al., 2016; Burke et al., 2018). However, to our knowledge, no reports have focused on the effects of the acceptable daily intake (ADI)-level exposure to neonicotinoids on mammalian brain function.

Since neonicotinoids have been regulated in many countries, including the European Union (EU), it is urgent to accumulate data on their toxicity studies regarding mammalian neurodevelopment from a precautionary principle viewpoint. In particular, two neonicotinoid pesticides [acetamiprid (ACE) and imidacloprid (IMI)] have the risk of affecting the developing human nervous system (EFSA, 2013). Furthermore, these neonicotinoids, like NIC, can invade the brain easily (Ford and Casida, 2006).

Our previous study focused on the hippocampal dentate gyrus (DG) (Saito et al., 2019). The DG of the mammalian brain is necessary for higher brain functions, such as learning and memory (Vivar and van Praag, 2013). Additionally, neurogenesis in the DG plays a functional role in the central nervous system and can be influenced by various environmental factors (Zhao et al., 2008).

In this study, we analyzed by adding NIC as a positive control whether low-level, chronic ACE and IMI exposure in early life with environmentally relevant concentrations could affect adult mice brain functions.

## 2. Materials and methods

### 2.1. Animals and treatments

**Figure 1:**
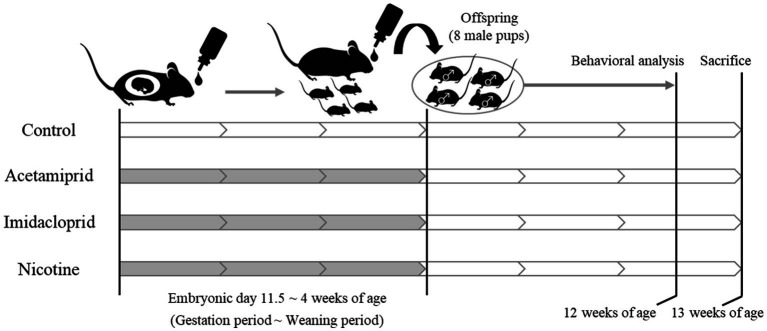
Schematic of neonicotinoids administration protocol. White: no treatment. Gray: Administration of neonicotinoids (or nicotine) by drinking water.

The experimental design is shown in Figure 1. Pregnant female C57BL/6 N mice at embryonic day 11 were purchased from Japan SLC (Shizuoka, Japan). Acetamiprid (ACE, Wako Pure Chemical Industries, Ltd. Osaka, Japan) and imidacloprid (IMI, Wako Pure Chemical Industries, Ltd.) were used as neonicotinoids, and (−)-nicotine (NIC, Wako Pure Chemical Industries, Ltd.) was used as a positive control. The mice were divided into four groups: (1) Control group; (2) ACE exposure group; (3) IMI exposure group; (4) NIC exposure group. The neonicotinoids concentration (0.01 mg/kg/day) of the exposure group was determined based on an acceptable daily intake (ADI) of 0.025 mg/kg/day for ACE and 0.06 mg/kg/day for IMI proposed by the European Food Safety Agency (EFSA) (EFSA, 2013). NIC is thought to be the effector of behavioral impairments because of its actions on nAChRs in the developing fetal brain (Pauly and Slotkin, 2008; Alkam et al., 2013a,b). The dose of NIC was 0.1 mg/kg/day, which induces behavioral effects of offspring in mice by administration during the prenatal period (Zhu et al., 2012). Females were exposed to ACE, IMI, and NIC from the gestation period (embryonic day 11.5) to maternal ablactation when pups were 4 weeks old. The control group received water. After maternal ablactation, eight male pups were randomly selected from each group and maintained until 13 weeks of age (these pups were selected from two dams in each group). Mice were maintained in a room at a constant temperature (24 ± 1°C) and humidity (60 ± 10%) room with a 12-h light/dark cycle and had free access to food and water. All animal care and experimental procedures were conducted following the Regulations for Animal Experiments and Related Activities at the National Institute of Health Sciences. The study was approved by the Animal Ethics Committee at the National Institute of Health Sciences (Permission #65-1).

### 2.2. Mouse behavioral test battery

The mouse behavioral test battery was conducted at 12 weeks of age. We performed a behavioral test battery that consisted of the open field (OF) test, the light/dark transition (LD) test, the elevated plus-maze (EP) test, the contextual/cued fear conditioning (FZ) test, and the pre-pulse inhibition (PPI) test. According to the previous report, these tests were performed (Tanemura et al., 2009; Juliandi et al., 2015; Furukawa et al., 2016; Saito et al., 2019) with some modifications. Experimental apparatus and image analysis software (ImageJ OF4, ImageJ LD2, ImageJ EP2, ImageJ FZ2, SR-9040) were obtained from O’Hara & Co., Ltd. ImageJ OF4, ImageJ LD2, ImageJ EP2, and ImageJ FZ2 were developed using the public domain ImageJ program. All experiments were performed sequentially using the same set of male mice (eight mice per group). The experimental tests were conducted between 13:30 and 16:30. The background noise level during the behavioral testing was about 50 dB. After each trial, the apparatus was cleaned with water and wiped.

Open field test: Locomotor activity was measured for 10 min using OF apparatus made of white plastic (50 × 50 × 40 [H] cm). An LED light system was positioned about 60 cm above the center of the field (50 lux at the center of the field). Behavior was measured with a charge-coupled device (CCD) camera positioned 50 cm above the center of the OF apparatus. Behavioral parameters were measured using ImageJ OF4.

Light/dark transition test: The apparatus used for the LD test consisted of a cage (21 × 42 × 25 [H] cm) divided into two chambers by a partition with an opening. One chamber was made of white plastic and was brightly illuminated (250 lux, light area), whereas the other chamber, made of black plastic, was dark (5 lux, dark area). Behavior was measured using a CCD camera positioned above each chamber. A mouse was placed in the dark area and allowed to move freely between the two chambers through the opening for 5 min. Behavioral parameters were measured using ImageJ LD2 and taken as indices of anxiety-related behavior.

Elevated plus maze test: The plus-shaped apparatus consisted of four arms (25 × 5 cm) connected to a central square area (5 × 5 cm). Two opposed arms were enclosed with 20 cm high transparent walls, and the other two were left open. The maze floor was made of white plastic and was elevated 50 cm above the room floor (10 lux at the center of the apparatus). Behavior was measured using a CCD camera positioned above each chamber. A mouse was placed in the central square area of the maze, facing one of the arms, and its behavior was recorded for 5 min. Behavioral parameters were measured using ImageJ EP2 to analyze anxiety-related behavior.

Contextual/cued fear conditioning test: The apparatus consisted of a conditioning chamber [or test chamber; 17 × 10 × 10 (H) cm] made of clear plastic with a ceiling. The chamber floor had stainless steel rods (2 mm diameter) spaced 5 mm apart for electric foot shock to the mouse. An LED light system was positioned 50 cm above the chamber floor (100 lux at the center of the floor). Behavior was measured with a CCD camera positioned 20 cm above the chamber ceiling. The white background noise level is set to about 50 dB in the soundproof box of white-colored wood. During the conditioning trial (day 1), mice were placed individually in the conditioning chamber and, after 90 s, they were given three-tone–shock pairings (30 s of tone at 65 dB, 10 KHz directly followed by 3 s of 0.1 mA electric shock), each separated by 120 s. Mice were then returned to their home cage. The next day (day 2), as a contextual fear test, they were returned to the conditioning chamber for 6 min without tone and shock. As a cued fear test on the third day (day 3), they were placed in a novel chamber (with a different design, lacking stainless steel rods, 50 lux at the center of the floor). After 3 min, the conditioning tone (with no shock) was presented for 3 min. ImageJ FZ2 measured the freezing response of the mice as a consecutive 2 s period of immobility. The freezing rate (%) was calculated as (time freezing/session time) × 100.

Pre-pulse inhibition test: The apparatus consists of a light source, sound system, and a startle measurement load cell. These are set into a soundproof box. The software for the operation of the apparatus and the data analysis is the SR-9040 (O’Hara & Co., Ltd., Tokyo, Japan). The white background noise level is set to 70 dB in the soundproof box. The mouse is put into a plastic cylinder and kept there for 90 s before the test. The test schedule consists of three blocks, and the total trial time is 30 min. Breakdown of each block is as follows: 80, 85, 90, 95, 100, 105, and 110 dB pulse × 3 (acclimation block), 120 dB pulse × 10 (acoustic startle response block). The combinations of pre-pulse are 80–120, 85–120, 90–120, 95–120, 100–120, 105–120 dB, with a delay of 100 ms × 6 (pre-pulse inhibition measurement block). These combinations were presented in a pseudorandom order, such that each trial type was presented once within a block. The inhibition ratio (%) of the startle response is calculated as follows: (1–pre-pulse [80, 85, 90, 95, 100, or 105 dB] startle response value/acoustic startle response value) × 100.

### 2.3. Tissue collection

After the mouse behavioral test battery, mice were euthanized under deep anesthesia by inhaling isoflurane at 13 weeks of age. The brains were surgically removed, fixed with methacarn solution (methanol:chloroform: acetic acid = 6:3:1), treated with ethanol and xylene, and embedded in paraffin, then sectioned into 10-μm slices at sagittal sections.

### 2.4. Immunohistochemical analysis

Paraffin-embedded sections were mounted on glass slides. Sections were gradually deparaffinized with xylene, rehydrated with ethanol (100, 95, 90, 80, and 70%), rinsed with distilled water, and then incubated with HistoVT One (Nacalai Tesque, Kyoto, Japan) at 90°C for 30 min. Next, sections were incubated with Blocking One (Nacalai Tesque) at 4°C for 1 h then incubated with primary antibodies at 4°C overnight. The following primary antibodies were used: mouse monoclonal anti-neuronal nuclei (NeuN; Merck Millipore; MAB377; diluted 1:500), goat polyclonal anti-SRY-related HMG-box 2 (SOX2; Santa Cruz Biotechnology; sc-17320; diluted 1:500), and goat polyclonal anti-glial fibrillary acidic protein (GFAP; Santa Cruz Biotechnology; sc-6170; diluted 1:500). After rinsing with phosphate-buffered saline (PBS), immunoreactive elements were visualized with Alexa Fluor 488-labeled anti-mouse and Alexa Fluor 594-labeled anti-goat secondary antibodies (Invitrogen; diluted 1:1,000) by treating at 4°C for 2 h. In addition, nuclei were stained with Hoechst 33342 (Nacalai Tesque; diluted 1:5,000). For all dilutions, the antibodies or Hoechst 33342 were added to a Blocking One and PBS mixture. Images were obtained with a BX51 microscope and analyzed with cellSens software (Olympus, Tokyo, Japan). For stained images of the DG, NeuN-positive (as a mature neuron marker), SOX2-positive (as a neural stem cell [NSC] marker), and GFAP-positive (as an astrocyte marker) cells were counted under the fluorescence microscope using a 20× objective. Based on our previous study (Saito et al., 2019), quantification of mature neurons (NeuN-immunoreactive cells), NSCs (SOX2-immunoreactive cells), and astrocytes (GFAP-immunoreactive cells) were also performed, with some modifications. To quantify each cell type, we examined eight brain sections per mouse, calculated positive cells of neural cell marker per section, and then compared it between each group. We used two offspring from each dam (in total, four mice per group). The 160 μm range of sagittal brain sections was used in this analysis.

### 2.5. Statistical analysis

Statistical analysis was performed using Dunnett’s test or Steel’s test after confirmation of normality and homoscedasticity with JMP 11 software (SAS Institute Inc., Cary, NC, United States). *p* < 0.05 was considered statistically significant.

## 3. Results

### 3.1. Mouse behavioral test battery

**Figure 2:**
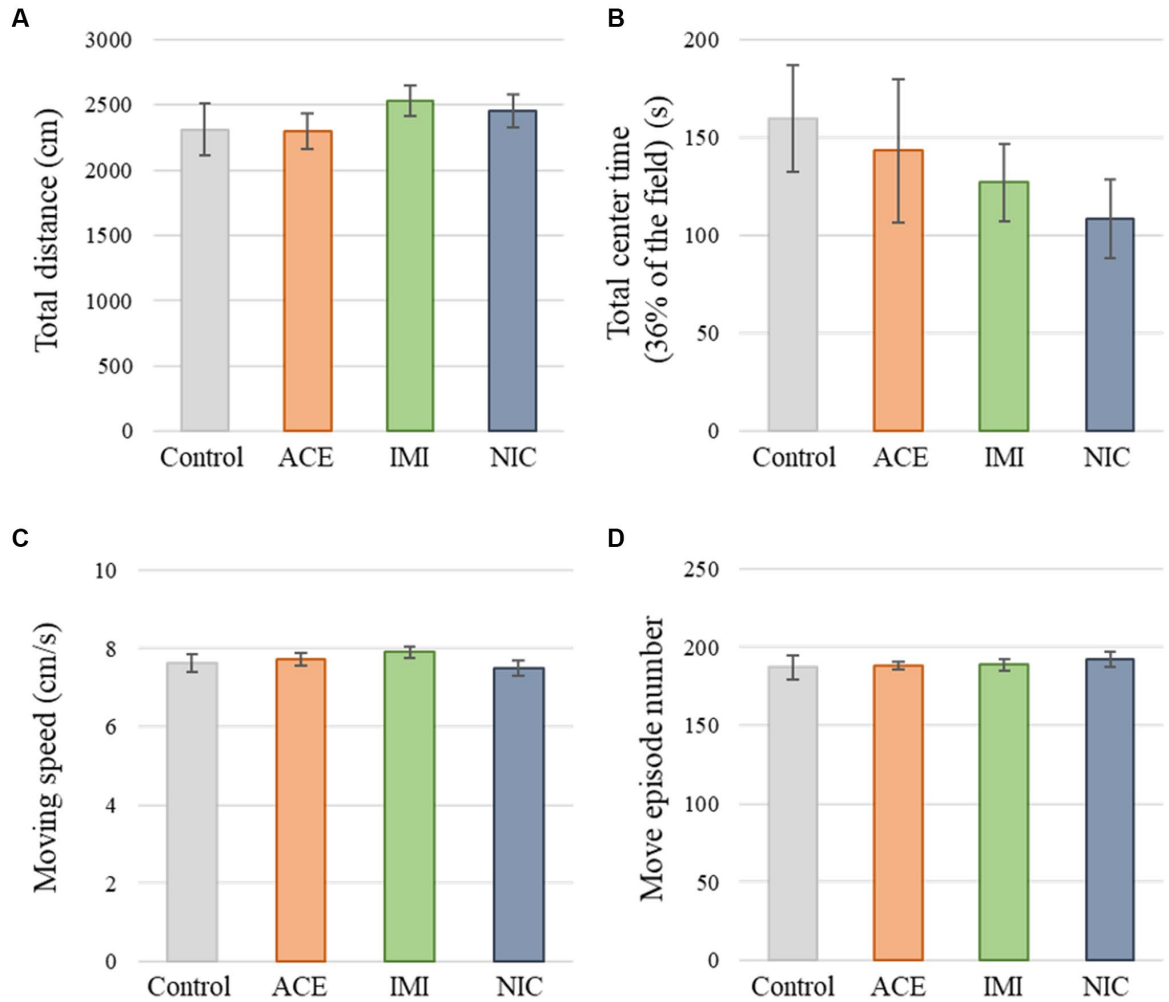
Results of the open field (OF) test. Representative scores of the OF test (total test time, 600 s) are shown. **(A)** Distance traveled (cm) during the test period. **(B)** Time spent in the center area (s). **(C)** Moving speed (cm/s) during movement. **(D)** Number of movements during the test period. Data are expressed as mean ± S.E. Data were tested statistically using Dunnett’s test or Steel’s test. *n* = 8 per group. ACE, acetamiprid exposure group; IMI, imidacloprid exposure group; NIC, nicotine exposure group.

**Figure 3:**
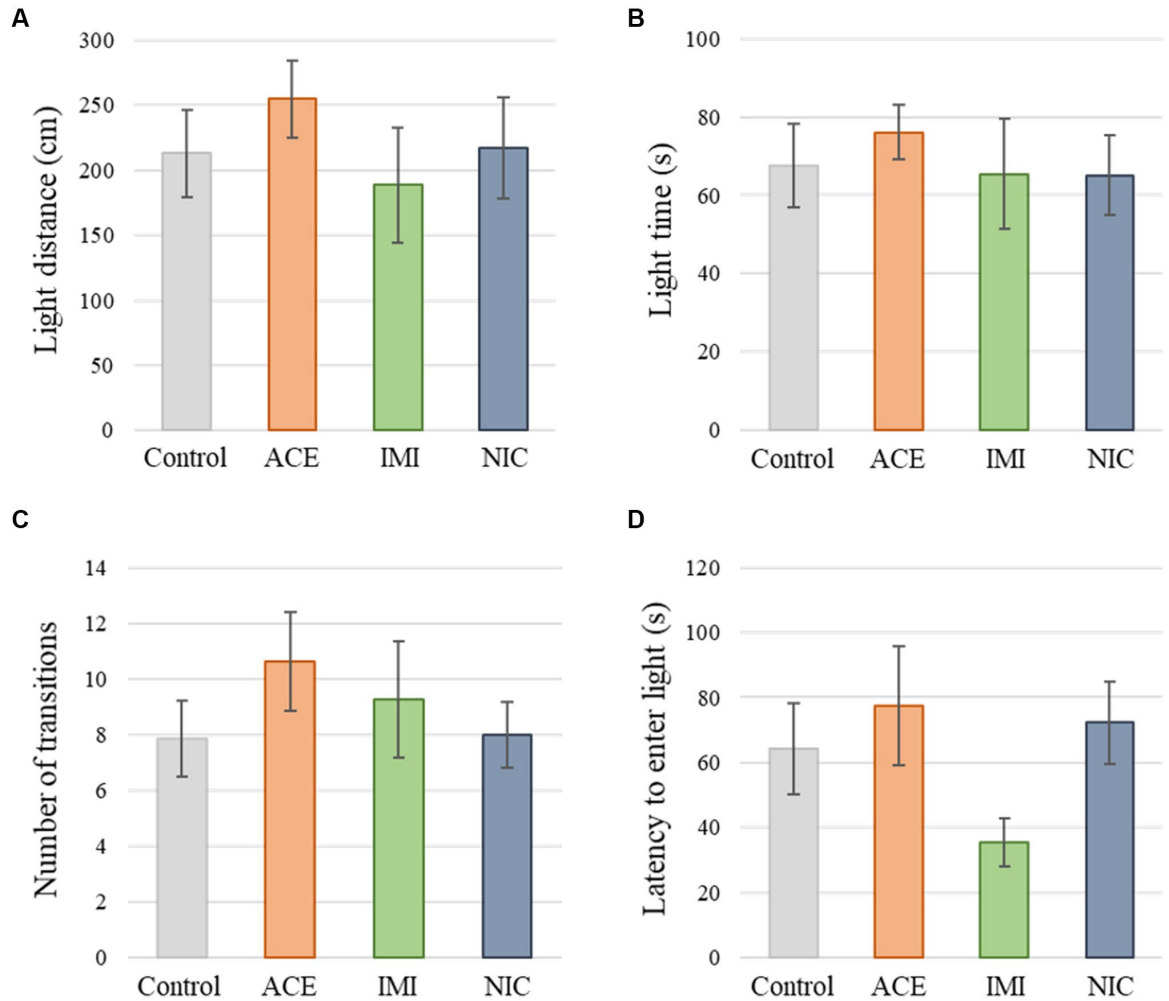
Results of the light/dark transition (LD) test. Representative scores of the LD test (total test time, 300 s) are shown. **(A)** Distance traveled in the light area (cm). **(B)** Time spent in the light area (s). **(C)** Number of transitions between the dark area and the light area. **(D)** Latency to enter the light area (s) for the first time. Data are expressed as mean ± S.E. Data were tested statistically using Dunnett’s test or Steel’s test. *n* = 8 per group. ACE, acetamiprid exposure group; IMI, imidacloprid exposure group; NIC, nicotine exposure group.

**Figure 4:**
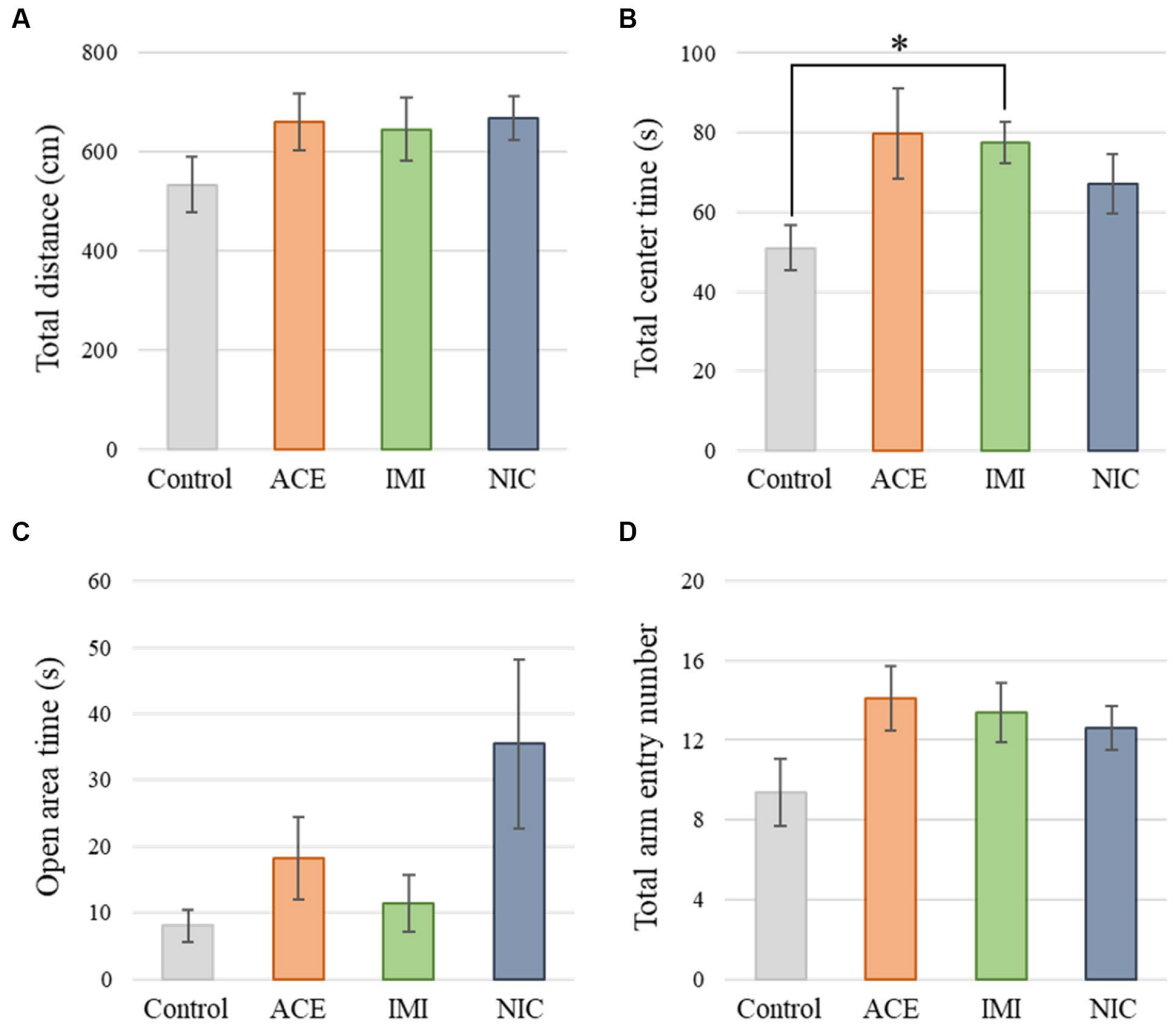
Results of the elevated plus-maze (EP) test. Representative scores of the EP test (total test time, 300 s) are shown. **(A)** Distance traveled (cm) during the test period. **(B)** Time spent in the center area (s). **(C)** Time spent in the open area (s). **(D)** Total entry number in arms. Data are expressed as mean ± S.E. Data were tested statistically using Dunnett’s test or Steel’s test. *p < 0.05 vs. the control group. *n* = 8 per group. ACE, acetamiprid exposure group; IMI, imidacloprid exposure group; NIC, nicotine exposure group.

**Figure 5:**
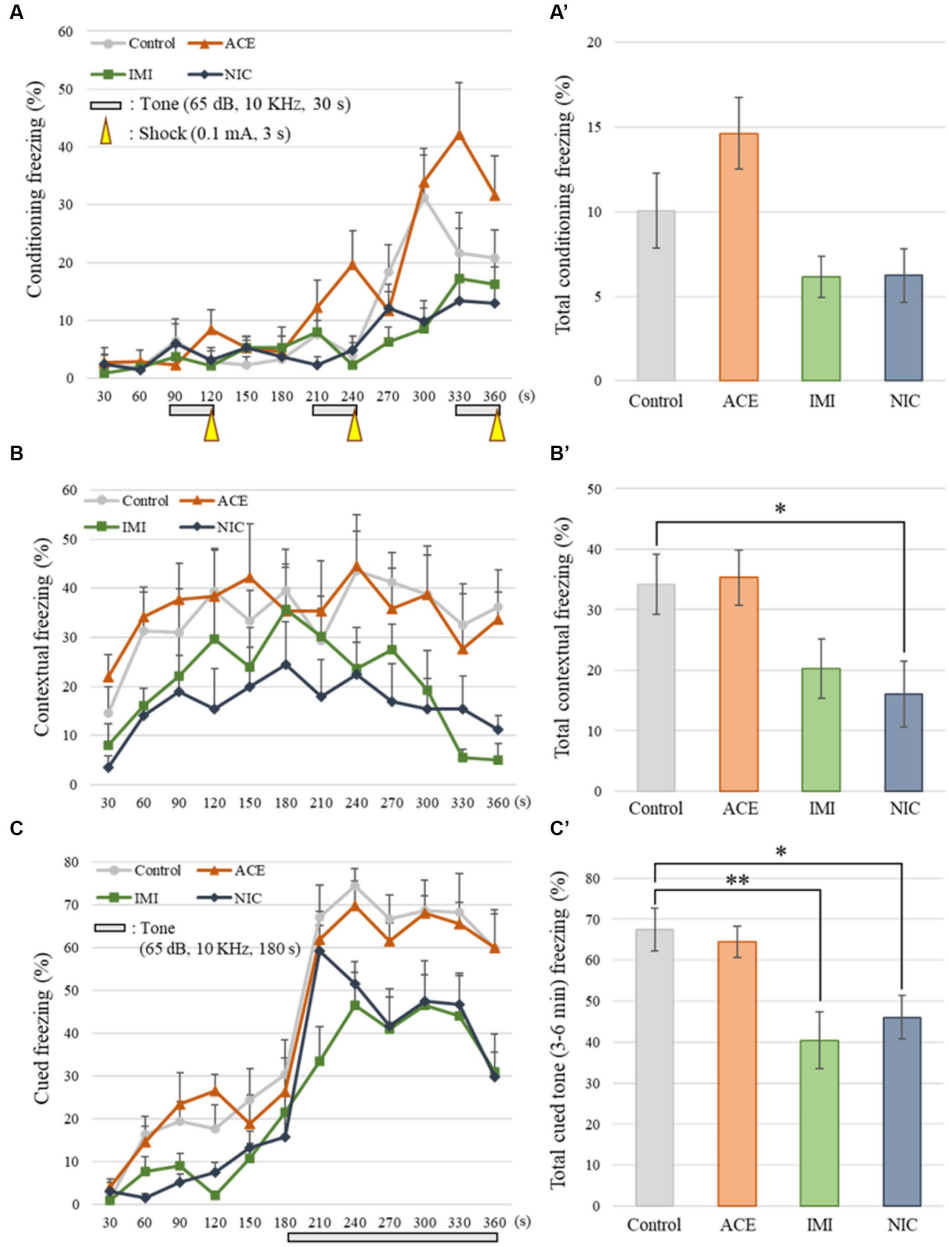
Results of the contextual/cued fear conditioning (FZ) test. **(A,A′)** The FZ test was conducted to analyze the effects of neonicotinoids on learning and contextual memory, i.e., place (test box) and sound (cued tone). The total conditioning time was 360 s. Three cycles consisting of a tone (30 s) and a mild foot shock (arrowheads: 0.1 mA, 3 s) were carried out for each mouse after it was allowed to explore the box freely for 90 s. The time course of the freezing scores (%) was plotted for the conditioning test, and the average total freezing scores (%) of control and neonicotinoid exposure groups in the conditioning test are shown. **(B,B′)** The contextual test was conducted to analyze the effects of neonicotinoids on place memory function. The total time of the test was 360 s. The time course of the freezing scores (%) was plotted for the contextual test, and the average total freezing scores (%) of control and neonicotinoid exposure groups in the contextual test are shown. **(C,C′)** The cued test was to analyze the effects of neonicotinoids on cued memory function. The total time of the test was 360 s. The time course of the freezing scores (%) was plotted for the cued test. The tone was presented to the mice during the later period of the test (180–360 s). The average freezing scores (%) of control and neonicotinoid exposure groups after the tone are presented. Data are expressed as mean ± S.E. Data were tested statistically using Dunnett’s test or Steel’s test. *p < 0.05, **p < 0.01 vs. the control group. *n* = 8 per group. ACE, acetamiprid exposure group; IMI, imidacloprid exposure group; NIC, nicotine exposure group.

**Figure 6:**
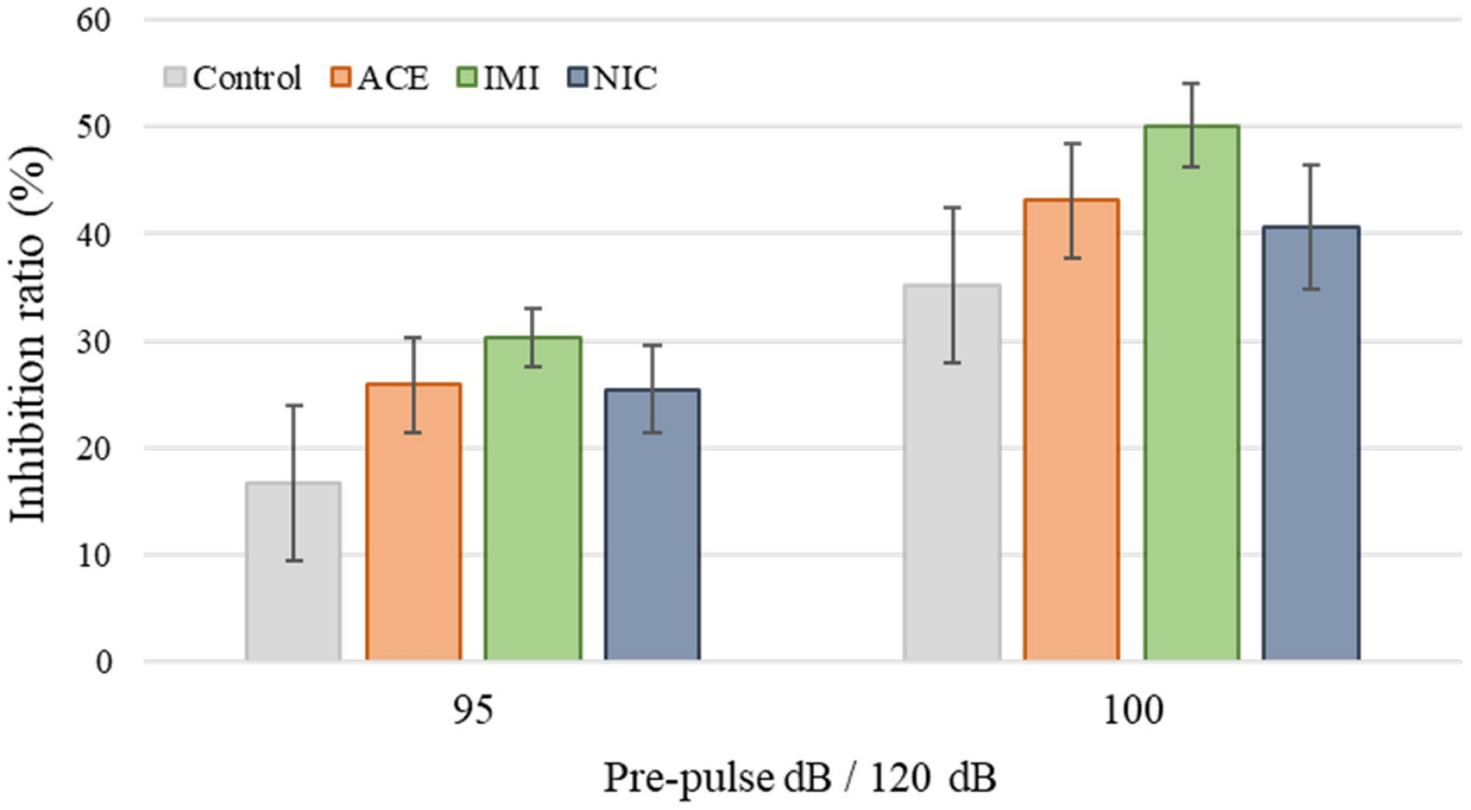
Results of the pre-pulse inhibition (PPI) test. Representative scores are shown. The inhibition (%) of the startle response to a 120-dB sound with pre-pulse sounds of 95 and 100 dB when compared to the response to a 120-dB sound without a pre-pulse sound. Data are expressed as mean ± S.E. Data were tested statistically using Dunnett’s test or Steel’s test. *n* = 8 per group. ACE, acetamiprid exposure group; IMI, imidacloprid exposure group; NIC, nicotine exposure group; ACE, acetamiprid exposure group; IMI, imidacloprid exposure group; NIC, nicotine exposure group.

**Figure 7:**
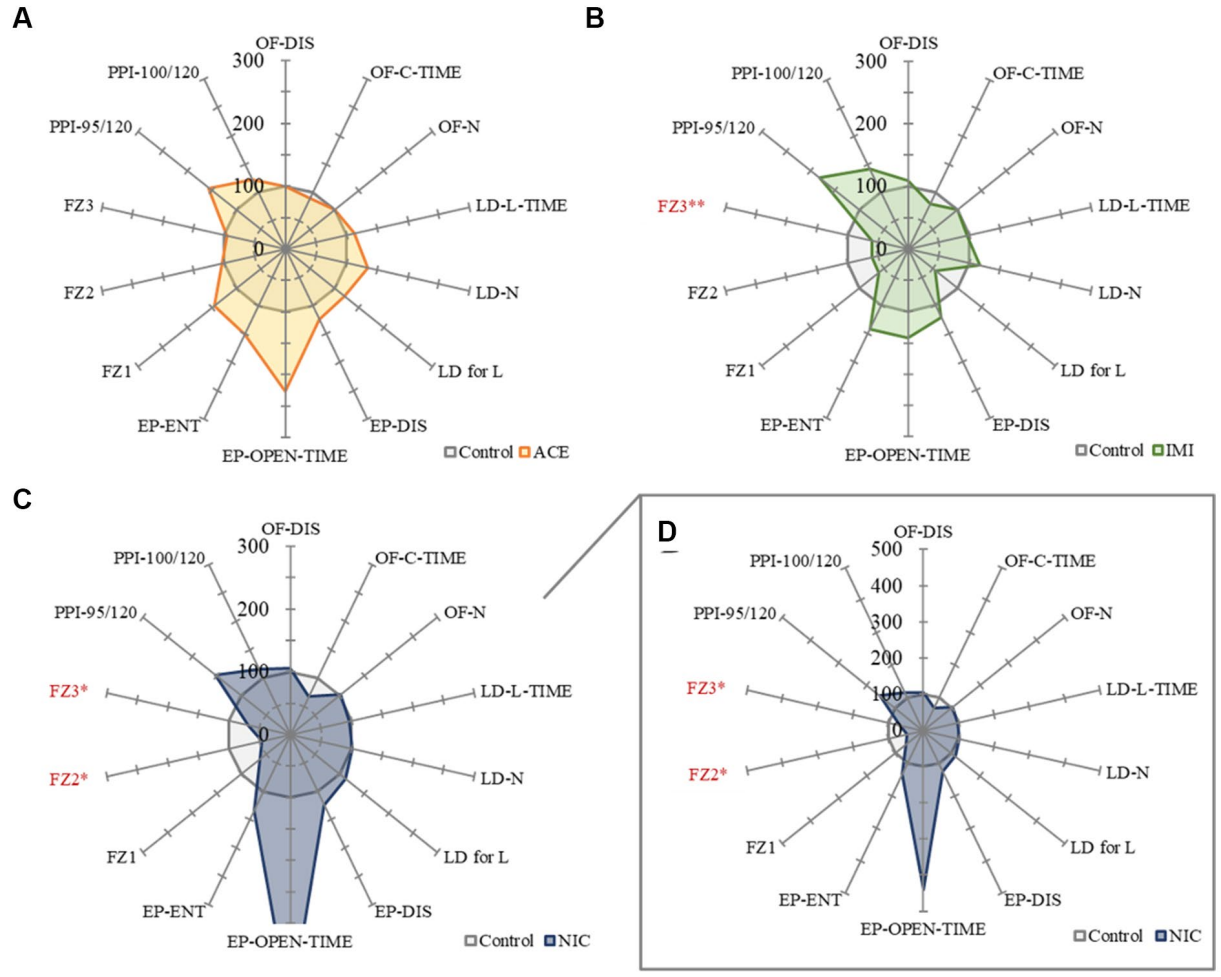
A summary of the mouse behavioral test battery. The main items of the mouse behavioral test battery were standardized by the average scores of control mice and shown by radar chart as the difference about the acetamiprid **(A)**, imidacloprid **(B)**, and nicotine **(C,D)** exposure groups against the control group (the average scores of control mice = 100%). Data are expressed as mean ± S.E. Data were tested statistically using Dunnett’s test or Steel’s test. *p < 0.05, **p < 0.01 vs. the control group. *n* = 8 per group. OF; OF-DIS, Total distance; OF-C-TIME, Total center time; OF-N: Move episode number. LD; LD-L-TIME, Light time; LD-N, Number of transitions; LD for L, Latency to enter light. EP; EP-DIS, Total distance; EP-OPEN-TIME, Open area time; EP-ENT, Total arm entry number. FZ; FZ1, Total conditioning freezing; FZ2, Total contextual freezing; FZ3, Total cued tone freezing. PPI; PPI-95/120, Pre-pulse 95 dB; PPI-100/120, Pre-pulse 100 dB. ACE, acetamiprid exposure group; IMI, imidacloprid exposure group; NIC, nicotine exposure group.

No significant differences were observed in the OF and LD test between ACE, IMI, and NIC exposure groups compared to the control group (Figures 2,3). In the EP test, the total center time was significantly increased in the IMI exposure group compared to the control group (Figure 4). As the conditioning cycles were repeated in the FZ test, freezing percentages increased in the ACE, IMI, NIC exposure, and control groups. This time-dependent change in the freezing rate confirmed that learning was established in each group (Figure 5A). In addition, no significant differences were observed in the freezing rate between the ACE, IMI, and NIC exposure groups and the control group (Figure 5A′). In the contextual fear test, the freezing rate was significantly lower in the NIC exposure group compared to the control group (Figures 5B,B′). Additionally, in the cued fear test, the freezing rate (after the cued tone) was significantly lower in the IMI and NIC exposure groups compared to the control group (Figures 5C,C′). Exposure to each chemical did not lead to any changes in the PPI test (Figure 6). The results of the behavioral test battery are summarized in Figure 7 and Table 1.

**Table 1:** Results of the mouse behavioral test battery in this study.

Test name	Behavioral parameters	Control	ACE	IMI	NIC
Open field	Total distance (cm)	2312.1 ± 197.4	2299.0 ± 136.9	2529.0 ± 115.0	2453.2 ± 123.5
	Total center time (36% of the field) (s)	159.8 ± 27.0	143.2 ± 36.9	127.0 ± 19.8	108.4 ± 20.2
	Average speed (cm/s)	3.9 ± 0.3	3.8 ± 0.2	4.2 ± 0.2	4.1 ± 0.2
	Moving speed (cm/s)	7.6 ± 0.2	7.7 ± 0.2	7.9 ± 0.1	7.5 ± 0.2
	Move episode number	187.0 ± 7.5	188.0 ± 2.4	188.9 ± 3.7	192.3 ± 5.1
	Total movement duration (s)	298.8 ± 19.0	296.6 ± 14.0	319.6 ± 9.1	326.3 ± 9.4
	Distance per movement (cm)	12.3 ± 0.8	12.2 ± 0.7	13.4 ± 0.5	12.9 ± 0.8
	Duration per movement (s)	1.6 ± 0.1	1.6 ± 0.1	1.7 ± 0.04	1.7 ± 0.1
Light/dark transition	Dark distance (cm)	864.5 ± 48.3	926.8 ± 40.0	906.4 ± 67.2	911.1 ± 46.7
	Light distance (cm)	212.9 ± 33.8	254.6 ± 29.4	188.5 ± 44.2	217.1 ± 38.5
	Dark time (s)	244.1 ± 9.7	235.8 ± 7.2	247.3 ± 12.9	245.6 ± 10.0
	Light time (s)	67.6 ± 10.8	76.2 ± 6.9	65.5 ± 14.1	65.1 ± 10.2
	Number of transitions	7.9 ± 1.4	10.6 ± 1.8	9.3 ± 2.1	8.0 ± 1.2
	Latency to enter light (s)	64.3 ± 14.0	77.4 ± 18.4	35.5 ± 7.4	72.3 ± 12.7
Elevated plus maze	Total distance (cm)	533.1 ± 55.7	659.4 ± 56.7	644.3 ± 63.8	666.9 ± 42.9
	Total center time (s)	50.9 ± 5.6	79.6 ± 11.4	77.4 ± 5.2*	67.1 ± 7.6
	Open area time (s)	8.1 ± 2.4	18.3 ± 6.2	11.4 ± 4.2	35.4 ± 12.7
	Close area time (s)	241.0 ± 7.4	202.1 ± 16.7	211.2 ± 7.0	197.5 ± 18.1
	Total arm entry number	9.4 ± 1.7	14.1 ± 1.6	13.4 ± 1.5	12.6 ± 1.1
Fear conditioning	Total conditioning freezing (%)	10.1 ± 2.2	14.6 ± 2.1	6.2 ± 1.2	6.2 ± 1.6
	Total contextual freezing (%)	34.2 ± 5.0	35.3 ± 4.5	20.3 ± 4.9	16.1 ± 5.4*
	Cued tone 3–4 min freezing (initial response) (%)	70.7 ± 4.4	65.8 ± 5.3	40.0 ± 7.1*	55.3 ± 5.1
	Total cued tone freezing (%)	67.5 ± 5.2	64.5 ± 3.8	40.4 ± 6.9**	46.0 ± 5.3*
Pre-pulse inhibition	Pre-pulse 95 dB (%)	16.6 ± 7.3	25.9 ± 4.5	30.3 ± 2.8	25.5 ± 4.0
	Pre-pulse 100 dB (%)	35.2 ± 7.3	43.2 ± 5.4	50.1 ± 3.9	40.6 ± 5.8

Data are expressed as mean ± S.E. Data were tested statistically using Dunnett’s test or Steel’s test. *p < 0.05, **p < 0.01 vs. the control group. *n* = 8 per group. ACE, acetamiprid exposure group; IMI, imidacloprid exposure group; NIC, nicotine exposure group.

### 3.2. Immunohistochemical analysis

**Figure 8:**
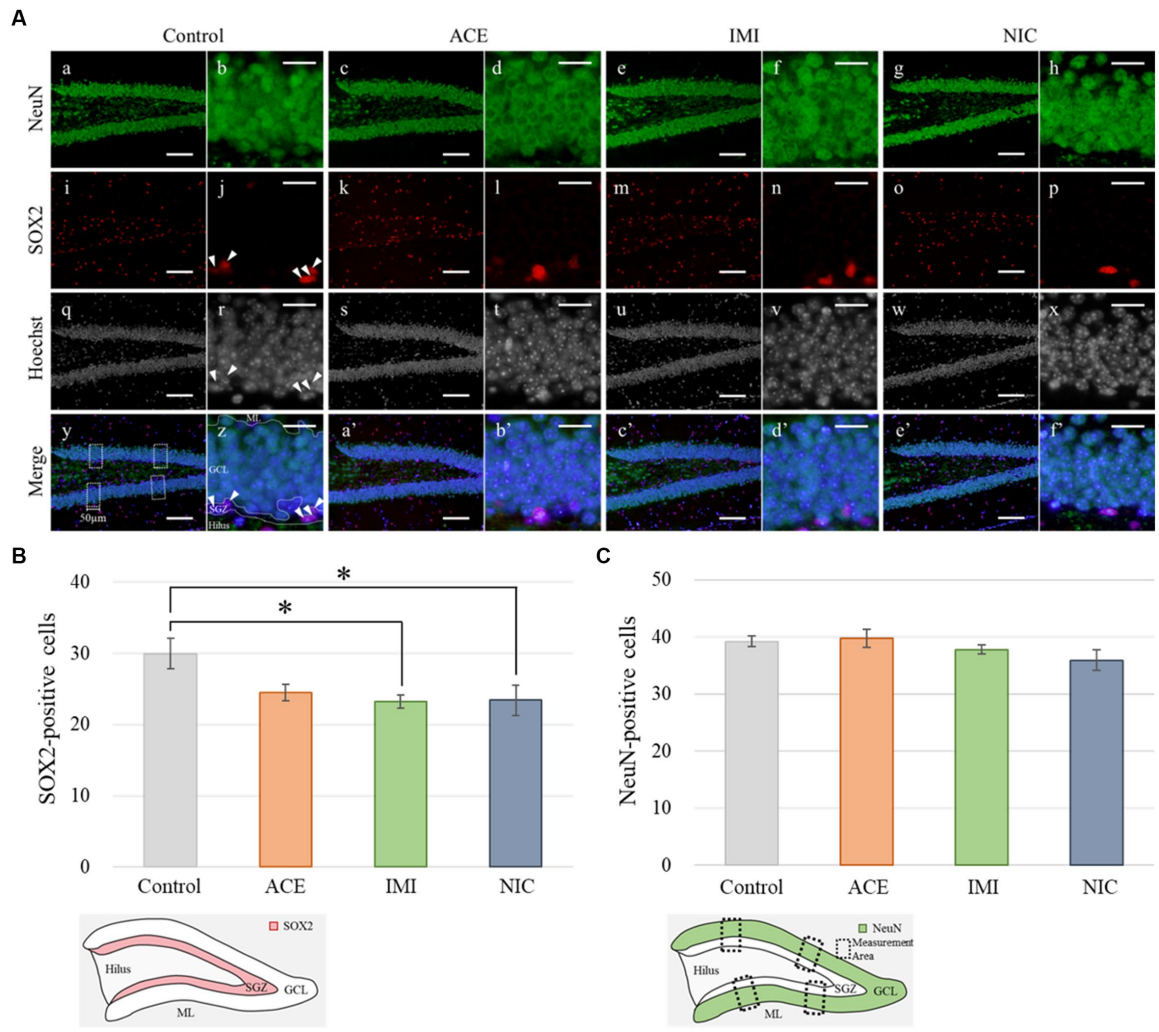
Immunohistochemical analysis with neuronal nuclei (NeuN) and SRY-related HMG-box 2 (Sox2). **(A)** NeuN and Sox2 immunohistochemistry of the dentate gyrus (DG) from 13-week-old (adult) male mice. The average of four sampling areas (50 μm in width; y) was used as the number of NeuN-positive cells. Mature neurons (NeuN-labeled) were observed in the granule cell layer (GCL) of the DG (a–h; green signals). The neural stem cells (SOX2-labeled) were observed in the subgranular zone (SGZ) of the DG (j, r, z; arrow heads). The nuclei were stained with Hoechst 33342. a, b, i, j, q, r, y, z: control; c, d, k, l, s, t, a’, b’: acetamiprid; e, f, m, n, u, v, c’, d’: imidacloprid; g, h, o, p, w, x, e’, f’; nicotine. Scale bars, 100 μm (a, c, e, g, i, k, m, o, q, s, u, w, y, a’, c’, e’), 20 μm (b, d, f, h, j, l, n, p, r, t, v, x, z, b’, d’, f’). **(B,C)** The number of NeuN- or SOX2-positive cells per brain section in each group (total number of NeuN- or SOX2-positive cells/number of brain sections examined per mouse). Data are expressed as mean ± S.E. Data were tested statistically using Dunnett’s test or Steel’s test. *p < 0.05 vs. the control group. *n* = 4 per group. ACE, acetamiprid exposure group; IMI, imidacloprid exposure group; NIC, nicotine exposure group; ML, molecular layer.

**Figure 9:**
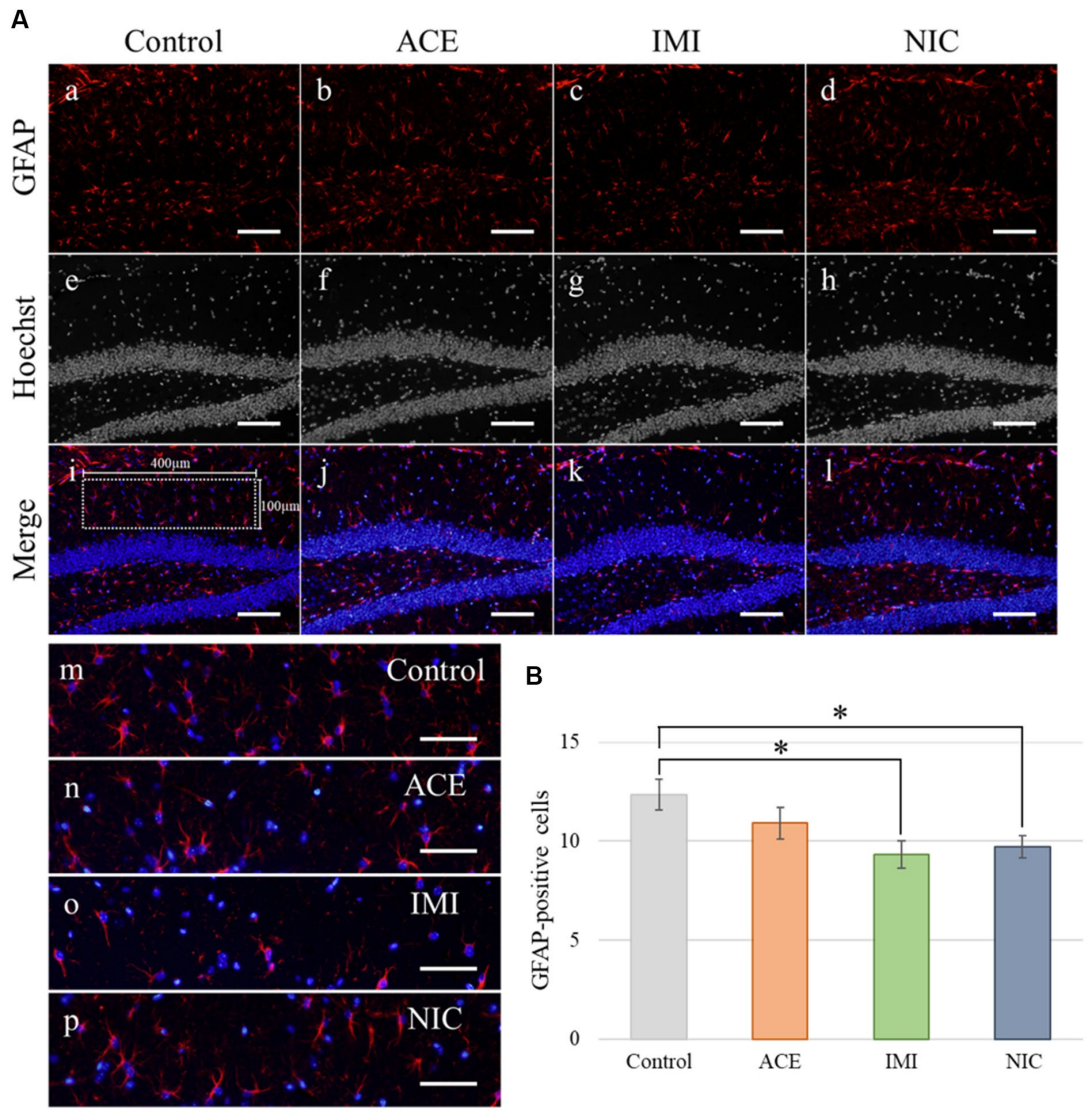
Immunohistochemical analysis with glial fibrillary acidic protein (GFAP). **(A)** GFAP immunohistochemistry of the dentate gyrus (DG) from 13-week-old (adult) male mice. The sampling areas in the molecular layer (400 × 100 μm/field; i) were used to calculate the number of GFAP-positive cells. **(B)** A decrease in astrocytes was observed in the molecular layer of the DG in the imidacloprid and nicotine exposure groups compared with the control group. Nuclei were stained with Hoechst 33342. a, e, i, and m: control; b, f, j, and n: acetamiprid; c, g, k, and o: imidacloprid; d, h, l, and p: nicotine. Scale bars, 100 μm (a–l), 50 μm (m-p). **(B)** The number of GFAP-positive cells per brain section in each group (total number of GFAP-positive cells/number of brain sections examined per mouse). Data are expressed as mean ± S.E. Data were tested statistically using Dunnett’s test. *p < 0.05 vs. the control group. *n* = 4 per group. ACE, acetamiprid exposure group; IMI, imidacloprid exposure group; NIC, nicotine exposure group.

In the IMI and NIC exposure groups, the number of SOX2-positive cells was significantly decreased compared to the control group (Control: 29.94 ± 2.10, ACE: 24.44 ± 1.16, IMI: 23.16 ± 0.93, NIC: 23.38 ± 2.12; *p* < 0.05; Figures 8A,B). For NeuN-positive cells, there were no significant differences in the ACE, IMI, and NIC exposure groups compared to the control group (Control: 39.21 ± 0.96, ACE: 39.73 ± 1.58, IMI: 37.76 ± 0.80, NIC: 35.90 ± 1.80; Figures 8A,C). In addition, immunohistochemical staining of GFAP demonstrated a decrease in GFAP-positive astrocytes in the molecular layer of the DG in the IMI and NIC exposure groups compared to the control group (Control: 12.34 ± 0.77, ACE: 10.91 ± 0.81, IMI: 9.31 ± 0.69, NIC: 9.72 ± 0.57; *p* < 0.05; Figure 9).

## 4. Discussion

This study investigated the effects of low-level, chronic neonicotinoids exposure in early life on the central nervous system of adult male mice. In addition, we augmented the data by adding nicotine as a positive control to the experimental design.

In the EP test, the total center time was significantly increased in the IMI exposure group compared to the control group. In the IMI exposure group, deviations were observed in the total center time of the OF test and latency to enter light of the LD test, even though the differences were not significant. In other words, we considered that deviations of anxiety-related behavior may cause some negative effects on motivation to explore in the EP.

No significant differences were observed in the FZ test between ACE, IMI, and NIC exposure groups and the control group in freezing rate during conditioning. However, in the contextual fear test, the freezing rate was significantly lower in the NIC exposure group compared to the control group. Additionally, in the cued fear test, the freezing rate (after the cued tone) of the IMI and NIC exposure groups was significantly lower than the control group. These results suggest that impairments in learning and memory (especially deviations in long-term memory) occur in the IMI and NIC exposure groups.

In the immunohistochemical analyses, in the IMI and NIC exposure groups, the number of SOX2-and GFAP-positive cells in the DG was significantly decreased compared to the control group. In mammals, NSCs that repeatedly self-renew in the early embryonic period acquire the ability to differentiate into neurons and then glial cells stepwise from mid to late embryonic period (Qian et al., 2000; Namihira et al., 2008). Especially, the late developmental stage to the postnatal period is a time when differentiation into astrocytes is active (Miller and Gauthier, 2007). Therefore, neural signaling disruption by IMI or NIC reduced the number of NSCs, eventually reducing the number of NSCs that can differentiate into astrocytes. Conversely, no significant difference between ACE, IMI, and NIC exposure groups and the control group in the number of mature neurons (NeuN-positive cells). In the previous study, although administration of trimethyltin that induces neurological dysfunction in mice causes the most marked loss of the granule cell layer and a marked decrease in NueN-positive cells in the hippocampal dentate gyrus within a few days, subsequent neurogenesis restores the cells to almost the same level as those in the control group (Ogita et al., 2005). Thus, in this study, we hypothesize that neonicotinoids cause the overproduction of neurons, and the regeneration of apparent mature neurons without functional maturation may occur in order to compensate for signal disturbance during brain formation. However, the effects of this disruption of division and survival on the construction of neural circuits are complex. Further studies, including the identification of cell types that are particularly affected during neural differentiation, are needed.

As we have quoted in a previous study, astrocytes are involved in the uptake of neurotransmitters released from neurons and the supply of energy to neurons (Kimelberg and Katz, 1985; Schousboe et al., 2004; Pierre and Pellerin, 2005). In addition, astrocytes are involved in the process of synaptogenesis (Song, H. et al., 2002; Song, H. J. et al., 2002; Hama et al., 2004) and assist in memory formation (Pabst et al., 2016). Similar to our previous findings (Saito et al., 2019), these key functions may be impaired due to the decrease in astrocytes. This result relates to the behavioral effects observed in the present study. In the developing brain, unbalanced specification of excitatory and inhibitory neurons leads to dysfunction of neural circuits and is a factor in various neurological disorders (Gatto and Broadie, 2010; Nelson and Valakh, 2015; Sawada et al., 2020). Therefore, the interference in the formation of neural circuits by IMI during the prenatal and developmental periods is one of the causes of the behavioral effects after maturation.

Cholinergic neurons in the brain are widely distributed in the central nervous system. In particular, the basal forebrain contains the nuclei of origin of cholinergic neurons in the central nervous system, projecting fibers to various regions such as the neocortex, hippocampus, and amygdala (Mesulam et al., 1983; Woolf, 1991; Ballinger et al., 2016). This acetylcholine-mediated neurotransmission is closely related to cognitive functions such as learning and memory (Conner et al., 2003; Mitsushima et al., 2013), and nAChRs modulate the neurobiological processes underlying hippocampal learning and memory (Kutlu and Gould, 2015). Although at lower levels than insects, mammalian nAChRs also have some affinity for neonicotinoids (Tomizawa and Casida, 2005). Therefore, the hippocampus was speculated to be one of the sites responsible for the effects of learning and memory induced by IMI and NIC exposure.

Contextual fear conditioning is highly dependent on the hippocampus (Phillips and LeDoux, 1992). It could reflect the effects on the hippocampus that the freezing rate in the contextual fear test showed a tendency to decrease in the IMI exposure group compared to the control groups, though there was no significant difference. The amygdala plays an important role in the associative learning of conditioned and unconditioned stimuli in classical fear conditioning (Wilensky et al., 2006). Therefore, it is necessary to consider the effect of the amygdala when regarding the results obtained in the cued fear test. In addition, FZ test is a coalitional learning using anxiety and/or fear caused by psychological stress. To evaluating the effects on pure memory, other analyses like maze learning must be combined.

In the ACE exposure group, no significant differences were observed in behavioral tests and immunohistochemical analysis compared to the control group. Namely, the effects of IMI on the central nervous system at these concentrations and administrations are considered to be higher than that of ACE. Although the detailed molecular mechanism of this difference is unknown, IMI may have more target molecules in the central nervous system compared with ACE. Additionally, in the behavioral test battery, one issue that must be considered is the degree of deviation from the control group mean. For example, the open area time of the EP test (important items as anxiety-related behavioral indicators) deviated substantially (more than 200%) from the mean value of the control group in the ACE and NIC exposure groups, even though there was no significant difference. This means there are several mice that anxiety levels deviate significantly. Therefore, bipolar effects (positive and negative bi-directional effects) are possible, and these may need to be considered as effects beyond individual differences in future studies.

Several studies have reported behavioral effects of nicotine exposure during the perinatal period that are inconsistent with our results. A previous study has demonstrated that gestational and/or perinatal exposure to nicotine is associated with the occurrence of emotional abnormalities including LD test, and showed the intensity of behavioral toxicity varies depending on the window of exposure (Alkam et al., 2013a). Anxiety-related and depression-like behaviors due to maternal nicotine exposure were also found only in female offspring and not detected in males (Liu et al., 2020). According to one study, behavioral testing after administration of nicotine resulted in a significant decrease in immobility time in the forced swim test in rats (Tizabi et al., 2010). On the other hand, a study in which nicotine was administered to adolescent rats showed a significant increase in immobility time in the forced swim test and depressive-like behavior in the sucrose preference test (Iñiguez et al., 2009). Thus, the effects induced by nicotine do not always show similar behavioral effects. In addition, different anxiogenic properties depending on the type of behavioral test were reported in a previous study. The results of correlations and factor analyses such as principal component analysis for OF, LD, EP tests, and other behavioral indicators of tasks suggest that task-specific basic behavioral characteristics and different control mechanisms for anxiety-related behaviors (Carola et al., 2002; Yilmazer-Hanke et al., 2003; Ramos, 2008). For these reasons, these non-uniform behavioral toxicities of nicotine are considered due to a variety of factors, including inter-strain differences, dose, duration or timing of administration, and weeks of age for behavioral testing.

The results of this study suggest that low-level neonicotinoid exposure in early life disrupted nAChRs-mediated neurotransmission, which had long-term effects on the brain structure and function of the litters. In particular, exposure to IMI was suspected to affect learning and memory and related neural circuit base. However, there are limitations to the results obtained with this experimental design. For example, increasing the number of test substances means increase in the number of experimental groups, and there are concerns about the effects of circadian variation on the behavioral patterns of the animals. This may make it difficult to detect the behavioral effects. Confirming dose dependence is also expected to make simultaneous comparison with multiple test substances difficult for the same reason. In addition to these trade-offs, effects of metabolites and sex differences also need to be considered. Moreover, exposure to pesticides occurs mainly through dermal, oral, and respiratory routes. Humans are directly exposed to pesticides in occupational, agricultural, and household activities and are indirectly exposed to pesticides via environmental media, including air, water, soil, and food (Tudi et al., 2022). Thus, it is necessary to consider the effects of exposure by various routes. As for transferability to the litter, one study revealed the presence of clothianidin, a type of neonicotinoid insecticide, and its five metabolites at approximately the same respective blood levels in both dams and fetuses (Ohno et al., 2020). In addition, another study showed that clothianidin concentrations in breast milk are also higher than those in the blood of dams (Shoda et al., 2023). In other words, similar effects may be expected for the test substances in this study. Therefore, it is necessary to accumulate data on an ongoing basis for the future, especially to carefully consider the potential effects on individuals during prenatal and early postnatal stages.

## Data availability statement

The original contributions presented in the study are included in the article/supplementary material, further inquiries can be directed to the corresponding author.

## Ethics statement

The animal study was approved by the Animal Ethics Committee at the National Institute of Health Sciences. The study was conducted in accordance with the local legislation and institutional requirements.

## Author contributions

HS and KT performed the conception and design of the experiments. HS and YF performed experiments. HS analyzed the data and wrote the paper. TS, SK, JK, and KT reviewed the study and edited the manuscript. All authors contributed to the article and approved the submitted version.

## Funding

This study was supported in part by the Health Sciences Research Grants from the Ministry of Health, Labour, and Welfare, Japan (H27-KAGAKU-IPPAN-007, H30-KAGAKU-IPPAN-003) and JSPS KAKENHI (Grant Number 19H01142).

## Conflict of interest

The authors declare that the research was conducted in the absence of any commercial or financial relationships that could be construed as a potential conflict of interest.

## Publisher’s note

All claims expressed in this article are solely those of the authors and do not necessarily represent those of their affiliated organizations, or those of the publisher, the editors and the reviewers. Any product that may be evaluated in this article, or claim that may be made by its manufacturer, is not guaranteed or endorsed by the publisher.
